# Aspirin-triggered Resolvin D1 ameliorates activation of the NLRP3 inflammasome *via* induction of autophagy in a rat model of neuropathic pain

**DOI:** 10.3389/fphar.2023.971136

**Published:** 2023-03-01

**Authors:** Yi-Hao Wang, Yu-Ru Tang, Xiao Gao, Nan-Nan Zhang, Qing-Qing Lv, Juan Liu, Yan Li

**Affiliations:** ^1^ Department of Pain Management, The Affiliated Hospital of Qingdao University, Qingdao, Shandong, China; ^2^ Department of Critical Care Medicine, The Affiliated Hospital of Qingdao University, Qingdao, Shandong, China; ^3^ Department of Geriatrics, Qingdao Mental Health Center, Qingdao, Shandong, China; ^4^ Department of Anesthesiology, The Affiliated Hospital of Qingdao University, Qingdao, Shandong, China; ^5^ Department of Obstetrics, The Affiliated Hospital of Qingdao University, Qingdao, Shandong, China; ^6^ Department of Anesthesiology, Shandong Provincial Maternal and Child Healthcare Hospital, Jinan, Shandong, China

**Keywords:** neuropathic pain, autophagy, Aspirin-triggered Resolvin D1, Nod-like receptor protein, microglia

## Abstract

**Background:** Several studies performed thus far indicate that neuroinflammation may be one of the mechanisms underlying the pathogenesis of neuropathic pain (NP). Autophagy, as an adaptive response, has been regarded as an active process of removing the inflammatory stimulus and restoring homeostatic balance. Resolution of inflammation is a biochemical process mediated by the so-called aspirin-triggered specialized proresolving lipid mediators (AT-SPMs), which are thought to exert protective effects in NP. Recent studies have proposed mechanisms in models of inflammatory disorders and showed a relationship between resolution of inflammation and autophagy. This study aimed to validate the functional effects of Aspirin-triggered Resolvin D1 (AT-RvD1) on *in vitro* and *in vivo* models of inflammation and to determine their roles in the regulation of autophagy and activation of the Nod-like receptor protein 3 (NLRP3) inflammasome signaling pathway.

**Methods:** An NP model was established using L5-6 spinal nerve ligation (SNL) and a model of tumor necrosis factor alpha (TNF-α)-stimulated primary microglia was established to evaluate the effect of SPMs. Western blotting was used to detect the level of NLRP3 inflammasomes complexes proteins (NLRP3, ASC, and Caspase-1) and autophagy-related proteins (LC3B, and Beclin1). Immunofluorescence staining was used to understand the autophagy and NLRP3 inflammasome activation process. The behavioral changes in rats were analyzed using paw withdrawal thresholds (PWT) and paw withdrawal latency (PWL) test.

**Results:** Our results showed that AT-SPMs significantly upregulated the activation of autophagy, which was characterized by an increase in the ratio of LC3B-II/I and accumulation of ATG5 and Beclin1. AT-RvD1 showed a dose-dependent decrease in the upregulated PWT and PWL induced by SNL and suppressed the expression of the NLRP3 inflammasome protein and the production of its corresponding downstream proinflammatory factors. Additionally, AT-RvD1 induced the activation of autophagy of the microglia and decreased the expression of the NLRP3 inflammasome protein and the accumulation of proinflammatory factors in TNF-ɑ-challenged microglia.

**Conclusion:** Thus, these results showed that AT-RvD1 may be a potential alternative therapeutic strategy for the prevention or treatment of NP by inhibition of the NLRP3 inflammasome signaling pathway by targeting the induction of autophagy.

## Introduction

Neuropathic pain (NP) is a chronic inflammatory condition caused by a lesion or dysfunction of the somatosensory system, and the symptoms of NP include a burning sensation, numbness, and allodynia (Finnerup et al., 2021). Previous studies have established the role of neuroinflammatory changes in the development of NP, and understanding the cellular and molecular biological mechanisms underlying NP is critical for developing an effective pharmacological therapy for NP (Giorgio et al., 2021; Mahmoud et al., 2021).

The Nod-like receptor (NLR) family protein NLRP3 inflammasome is a component of the innate immune system, and it can recognize several pathogens and environmental and host-derived factors. Upon appropriate stimulation, the NLRP3 assembles the apoptosis-associated speck-like protein (ASC) and pro-Caspase-1 required for inducing the expression of the proinflammatory cytokines (Mangan et al., 2018; Swanson et al., 2019). Recent studies (Saresella et al., 2016; McKenzie et al., 2018) indicate that persistent activation of the NLRP3 inflammasome complex and its by-products is responsible for triggering the neuro-inflammatory changes involved in neurodegenerative diseases (Saresella et al., 2016; McKenzie et al., 2018), also in the progression of NP (Ren et al., 2021; Sun et al., 2021). Thus, an increased incidence of neuroinflammatory diseases in the patient population warrants additional studies for establishing an optimal therapeutic intervention.

Autophagy is a ubiquitous cytoprotective process that plays a regulatory role in every aspect of cellular biology from pathogen recognition to cytokine release, inflammasome activation, and nervous system homeostasis (Li et al., 2021; Vidal et al., 2021). Previous studies have highlighted the potential impact of dysfunction of autophagy in the pathogenesis of various conditions across most neurodegenerative diseases (Chu, 2019; Liu et al., 2019; Park et al., 2020). Autophagic process is proposed as a latent therapeutic target for the stage of NP, which represents a relatively new area of study (Cosin-Roger et al., 2017a). However, the precise mechanisms underlying the relationship between the autophagic process and neuroinflammatory changes have not been completely elucidated thus far. Recent studies have shown that NLRP3 inflammasome serves as the convergence point for many of the upstream stimuli and pathways, and the activity of NLRP3 was regulated by the autophagic process (Mehto et al., 2019; La Rosa et al., 2022). Therefore, regulation of autophagy-dependent NLRP3 inflammasome mediation may be an ideal therapeutic strategy for NP. This study aimed to determine the regulation of intracellular autophagy pathways involved in neuroinflammation.

Aspirin-triggered specialized proresolving mediators (AT-SPMs) are highly potent mediators with potent proresolving actions that trigger the biosynthesis of endogenous mediators that actively promote the termination of inflammatory responses (Chen et al., 2018; Chiang and Serhan, 2020). Preliminary experimental evidence including ours indicates that AT-SPMs may be used for alleviating NP hypersensitivity (Fattori et al., 2020; Wang et al., 2020; Leuti et al., 2021; Wang et al., 2021). An association exists between the regulation of inflammation resolution and autophagic mechanisms in chronic inflammatory response (Recchiuti et al., 2020). In this study, we aimed to further increase the understanding of the mechanisms involved in the initiation and propagation of the neuroinflammatory response in NP (Recchiuti et al., 2020; Wang et al., 2021). Thus, we established an *in vitro* and *in vivo* model of inflammation to further assess the anti-nociceptive effectiveness of AT-SPMs and explored the potential mechanisms involved in the NLRP3 inflammasome and autophagic process.

## Materials and methods

### Animal preparation

Male adult Sprague–Dawley (SD) rats weighing 250–350 g were obtained from the Experimental Animal Center of the Medical College of Shandong University (Shandong, China). The rats were housed in separate cages (5–6 per cage) and were fed in a specific pathogen-free animal facility with standard lab food and water *ad libitum* under a 6:00–18:00-h light cycle at controlled room (22°C ± 2°C) temperature and 50%–60% humidity. All experimental animal procedures were followed and approved by the Experimental Animal Care and Use Committee of the Institute of Qingdao University.

### Establishment of an NP model and drug administration

The NP model was established *via* unilateral L5-6 spinal nerve ligation (SNL) according to the method suggested by Ye and Savelieva (2015) (Ye et al., 2015). Briefly, the SD rats were anesthetized intraperitoneally with 1% sodium pentobarbital (Nembutal, 50 mg/kg), the left L5-6 nerve was exposed by blunt dissection, and transected distal to the ligation using a 5–0 silk thread. To examine the therapeutic and mechanistic effect of AT-RvD1 on the established spinal never injury, the animals were randomly administrated to different doses of AT-RvD1 (Cayman Chemical, 10 or 100 ng per rat per day, intrathecally) and 3-methyladenine (3-MA; MedChemExpress, HY-19312, 15 mg/kg/d, intraperitoneally) divided into different treatment groups after SNL surgery. The rats were injected with different doses of AT-RvD1 or 3-MA for the first three consecutive days after surgery according to our previous studies (Wang et al., 2020; Wang et al., 2021). On Day 7 after SNL, cervical dislocation was performed when the rats were lightly anesthetized with pentobarbitone (120 mg/kg, intraperitoneally). The ipsilateral dorsal horn of spinal cord for each rat was removed and collected for further experimental analysis.

### Sensory sensitivity testing

Assessment of paw withdrawal thresholds (PWT) to mechanical stimulus and paw withdrawal latency (PWL) to thermal stimulus was performed to evaluate the pain-related behaviors. The von Frey (Stoelting, United States) withdrawal test was used to examine the mechanical sensitivity the “up-down” method (Chaplan et al., 1994) responsed to punctuate the mechanical stimuli on the surface of the hind paw of the rats. The degree of PWL was measured following Hargreaves’ test (Ugo Basile, Varese, Italy) (Hargreaves et al., 1988). The preoperative day 1 of SNL and postoperative interval days from 1 to 21 were the testing days for the long observation. Before testing the sensitivity, all rats were acclimatized to separate chambers for at least 30 min. The procedure was repeated in each rat at least 2–3 times with a 5-min interval, and the average data of paw withdrawal threshold was calculated. The positive reaction was defined as withdrawal, shaking, or licking of the hind limb. All behavioral assays were conducted by at least two investigators who were blinded to the experimental conditions.

### Cell culture

Newborn rats (1- to 2-day-old) were killed by decapitation, and their cerebral cortices were rapidly isolated under sterile conditions. The brain tissues were homogenized and placed in a Petri dish containing Dulbecco’s modified Eagle medium (nutrient mixture F-12, DMEM F1/2) and were digested using 0.25% pancreatic enzymes. After centrifuging at 220 × *g* for 5 min and filtration using a nylon screen, cell pellets were cultivated at 37°C under humidified 5% CO_2_/95% air for 14 days. The purity of the cell suspension was examined by staining with OX42 (mouse anti-CD11b, 1: 100), and cells showing OX42 immunoreactivity were determined positive. The following experiments were performed after culturing for 2–4 days.

The cultured cells were stimulated with TNF-α (20 ng/mL) for 24 h before harvesting the cells. Then, the cells were incubated with different concentrations of AT-RvD1 (1 and 10 nM), MCC950 (a selective inhibitor of NLRP3, MedChemExpress, HY-12815, 10 µM), 3-MA (5 mM), or bafilomycin A1 (BafA1; an autophagosome-lysosome fusion inhibitor, MedChemExpress, HY- 100558, 20 nM) before stimulation with TNF-α.

### Immunofluorescence

Samples were fixed with 4% paraformaldehyde for 30 min, and blocked with 3% bovine serum albumin (BSA) at room temperature for 1 h, and then incubated overnight at 4°C with the indicated primary antibody (LC3B, Cell Signaling CST, 43566; ATG5, Proteintech, 10181-2-AP; NLRP3, Proteintech, 19771-1-AP; ASC, ABclonal, A16672). Subsequently, the cells were exposed to a fluorescein isothiocyanate (FITC)-conjugated or Cy3-conjugated secondary antibody (1:200, Jackson ImmunoResearch Laboratories) for 1 h (1:500) and were counterstained with 4′,6-diamidino-2-phenylindole (DAPI; Roche, 10236276001) nuclear stain and incubated for 5 min in the dark.

Then, the cells were sealed with a sealing liquid containing an anti-fluorescence agent (Thermo Fisher Scientific, Waltham, MA, United States), and the images were captured using a Leica MD4000B fluorescence microscope (Leica, Germany).

### Autophagic flux analysis

To detect autophagic flux, microglial cells pretreating with TNF-α were transfected with an mRFP-GFP-LC3B adenovirus (Hanbio Co. Ltd., Shanghai, China) with or without 10 nM AT-RvD1. The fluorescence images were observed and staining was performed using the Leica Microsystems TCS SP8 confocal microscope (Olympus Fluoview™; FV1000, Japan). The colocalized red and green fluorescence puncta were calculated to show the activation of autophagy.

### Western blot analysis

The concentrations of protein samples were measured using the enhanced bicinchoninic acid (BCA) protein assay kit (Beyotime, Beijing, China). The cells were homogenized and lysed using the radioimmunoprecipitation assay (RIPA) buffer, and then, were centrifuged at 12,000 rpm for 15 min at 4°C to obtain the total protein. The lysates were electrophoresed using 12% sodium dodecyl sulfate-polyacrylamide gel electrophoresis (SDS PAGE, Thermo Fisher Scientific), and the bands were electrotransferred onto a 0.2-µm polyvinylidene fluoride (PVDF) membrane (GE Healthcare Biosciences, United States) overnight at 4°C. After blocking with 5% non-fat dry milk for 1 h at room temperature, the membranes were subsequently probed with the respective primary antibodies against NLRP3 (Proteintech, 19771-1-AP), ASC (ABclonal, A16672), Caspase-1 (Proteintech, 22915-1-AP), Beclin1 (Cell Signaling Technology, 3495), LC3B (Cell Signaling Technology, 43566), p-ERK1/2 (abcam, ab201015) and p-AKT (Cell Signaling Technology, 4060) in Tris buffered saline (TBS). Then, the membranes were incubated using the corresponding goat anti-rabbit immunoglobulin G (IgG) horseradish peroxidase-conjugated secondary antibodies (1:2000; Beyotim e Biotechnology) at room temperature for 1 h. The target protein bands of bound antibodies were examined using enhanced chemiluminescence (ECL; GE Healthcare Biosciences, United States) reagents in accordance with the instructions of the manufacturer. The positive bands were visualized using a CCD camera (LAS-3000 luminescence image analyzer, Fuji Film, Tokyo, Japan) and were analyzed using the Image-Pro Plus software version 6.0 (Media Cybernetics).

### Enzyme-linked immunosorbent assay analysis

The levels of interleukin 1β (IL-1β), IL-18, and TNF-α were measured using the enzyme-linked immunosorbent assay (ELISA) (R&D Systems) kit according to the manufacturer’s instructions. The optical density (OD) was measured at a wavelength of 450 nm using a plate reader (Thermo Fisher Scientific). The analytical sensitivity for ELISA kits of inflammatory cytokines is less than 5 pg/ml according to the user guide.

### The Caspase-1 activity test

The caspase-1 activity was assayed by using caspase-1 activity assay kit (Beyotime, China) according to the manufacturer’s instructions. The absorbance was measured at a wavelength of 405 nm.

### Statistical analysis

Experimental data depicted in graphs were expressed as mean ± standard error of mean (SEM) of at least three independent experiments. Differences between groups were determined using Student’s t*-*test or one- or two-way analysis of variance (ANOVA). A *p*-value < .05 was considered to be statistically different. A *p*-value < .01 was considered to be a statistically significant difference. The statistical analysis was performed using SPSS Statistics 20.0 (Version X; IBM, Armonk, NY, United States).

## Results

### AT-RvD1 ameliorated the SNL-Induced hyperalgesia behavior and inhibited the activation of proinflammatory cytokines

**FIGURE 1:**
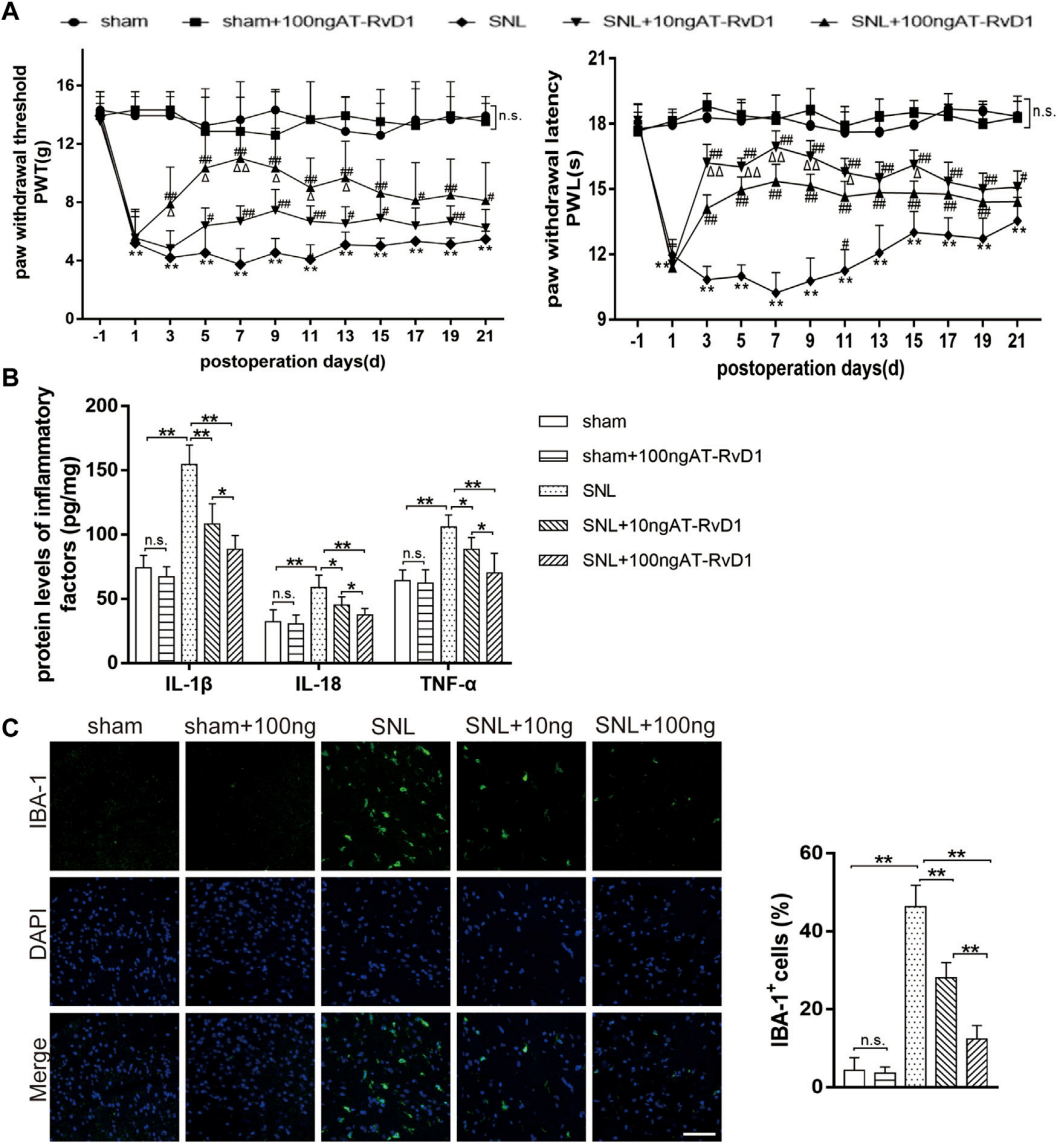
The effect of AT-RvD1 administration on the level of proinflammatory cytokines and spinal nerve ligation-induced mechanical hyperalgesia in rats **(A)** Time course analysis of behavior demonstrating the effect of AT-RvD1 on spinal nerve ligation (SNL)-induced mechanical allodynia (*n* = 6/group). The paw withdrawal threshold (PWT) and paw withdrawal latency (PWL) were measured from the day before surgery to postoperative day 21. **(B)** Dose-dependent effect of AT-RvD1 on the expression of interleukin 1β (IL-1β), IL-18, and tumor necrosis factor alpha (TNF-ɑ) was examined using enzyme-linked immunosorbent assay (ELISA) (*n* = 6/group). **(C)** Dose-dependent effect of AT-RvD1 on the expression of Iba1 in microglia in the spinal cord dorsal horn (*n* = 3/group) (scar bars = 60 µm for figures). Data were expressed as mean ± standard error of mean (SEM). *p < .05 and **p < .01 compared with the sham group; ^#^ *p* < .05 and ^##^ *p* < .01 compared with the SNL group; ^△^ *p* < .05 compared with the 10 ng AT-RvD1group; n. s. means no significant difference between independent experiments.

To explore the role of AT-RvD1 in behaviors associated with NP, we performed behavioral testings before surgery and on the 1st, 3rd, 5th, 7th, 9th, 11th, 13th, 15th, 17th, 19th, and 21st day after surgery (Figure 1A). After L5-6 SNL, the paw withdrawal threshold (PWT) and paw withdrawal latency (PWL) of the treatment group were significantly lower than those of the sham group (Figure 1A). AT-RvD1 increased the PWT and PWL in a dose-dependent manner. According to the long observational PWT and PWL results, we chose the 7th day after surgery to perform the following experiments because of the strongest hyperalgesia behavior and optimal analgesic effect. Then, we measured the production of different proinflammatory cytokines (IL-18, IL-1β, and TNF-ɑ) after SNL (Figure 1B). Results of ELISA showed that the levels of proinflammatory cytokines in the spinal cord dorsal horn in the SNL group were significantly higher than those in the sham group (Figure 1B). Intraperitoneal injection of AT-RvD1 decreased the SNL-induced expression of IL-18, IL-1β, and TNF-ɑ in a dose-dependent manner. As known, microglia are the resident cells in the nervous system involved in neuroinflammation. Therefore, we checked the microglia activation in the spinal cord dorsal horn of different groups. The results showed that the expressions of Iba1 in microglia in the SNL group were significantly increased compared with the sham with or without AT-RvD1 group. And the AT-RvD1 treatment could decreased the expressions of Iba1 in the SNL group dose-dependently (Figure 1C).

### The role of AT-RvD1 in the progression of autophagy in rats with SNL-induced NP

**FIGURE 2:**
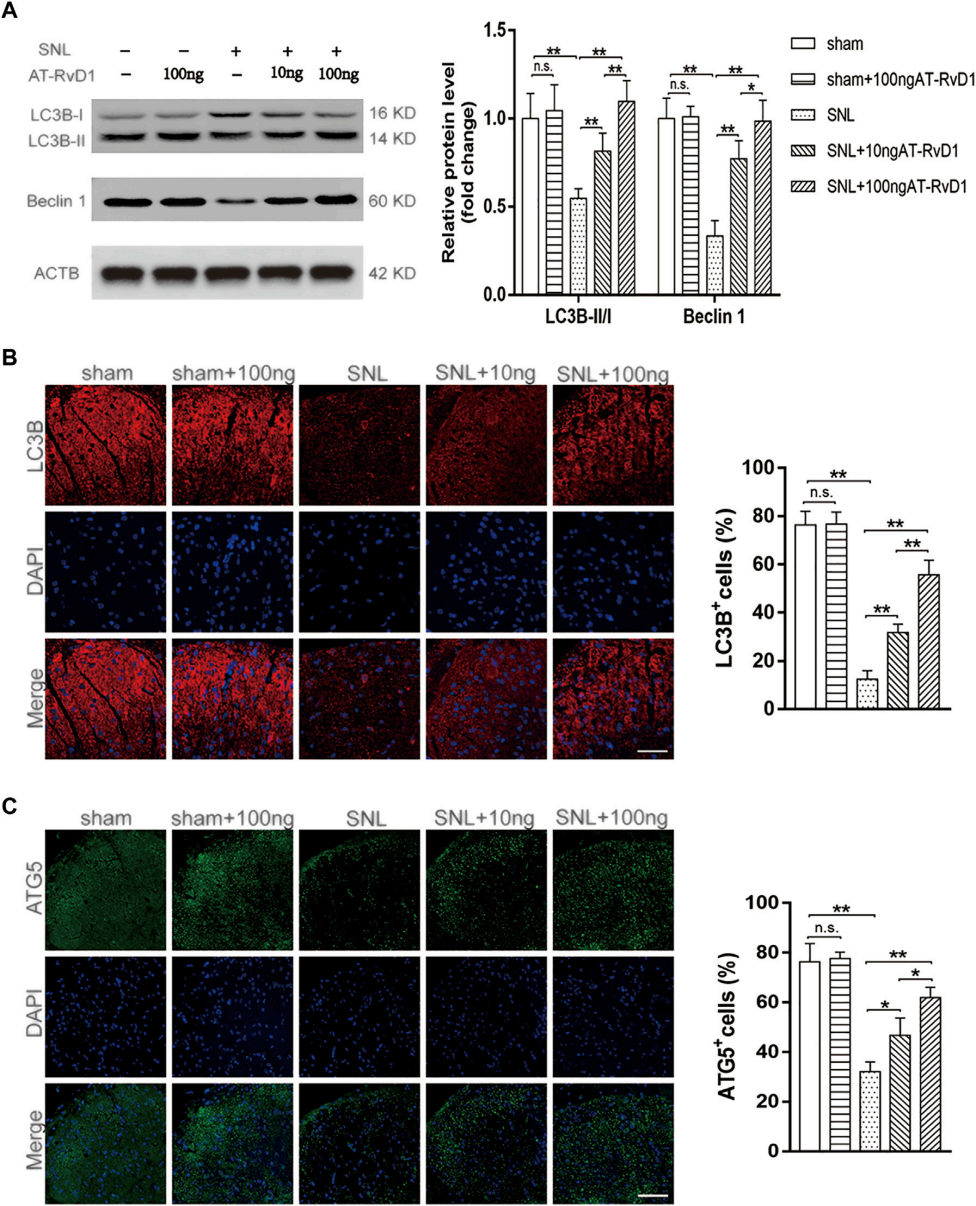
Effect of administration of AT-RvD1 on activation of autophagy in a rat model of neuropathic pain **(A)** Representative photomicrographs of western blot bands and densitometric analysis for quantifying the expression of proteins Beclin1, LC3B-II, and LC3B-I in different groups (*n* = 5/group). **(B)** Immunostaining of LC3B puncta was visualized using fluorescence microscopy (*n* = 3/group) (scar bars = 60 µm for figures). **(C)** Immunostaining of ATG5 puncta was visualized using fluorescence microscopy (*n* = 3/group) (scar bars = 60 µm for figures). Data were expressed as mean ± standard error of mean (SEM). ∗*p* < .05. ∗∗*p* < .01. n. s. means no significant difference between independent experiments.

To analyze the function of autophagy in NP, we performed immunoblot staining of autophagy-related markers harvested from the spinal cord dorsal horns of the NP rats. The expression of the autophagy-related proteins (Beclin1) and the autophagosome LC3B-II/I ratio were maintained at a high level in the sham group, but those downregulated in rats with SNL-induced NP (Figure 2A). Results of immunoblotting analysis showed that administration of AT-RvD1 resulted in a dose-dependent increase in the expression of LC3BII/LC3BI and Beclin1 in rat spinal cords (Figure 2A). Additionally, results of immunofluorescence staining (Figure 2B) showed that the rats in the NP group had a lower number of LC3 immunopositive cells in rat spinal cords than the rats in the sham group AT-RvD1 administration increased the LC3-positive puncta following SNL. Autophagy-related (Atg) genes are indispensable in the process of autophagy. Therefore, we also examined the ATG5 protein with immunofluorescence assay (Figure 2B). The results were similar with LC3 protein.

### The protective effects of autophagy in TNF-α-stimulated microglial status mediated by AT-RvD1

**FIGURE 3:**
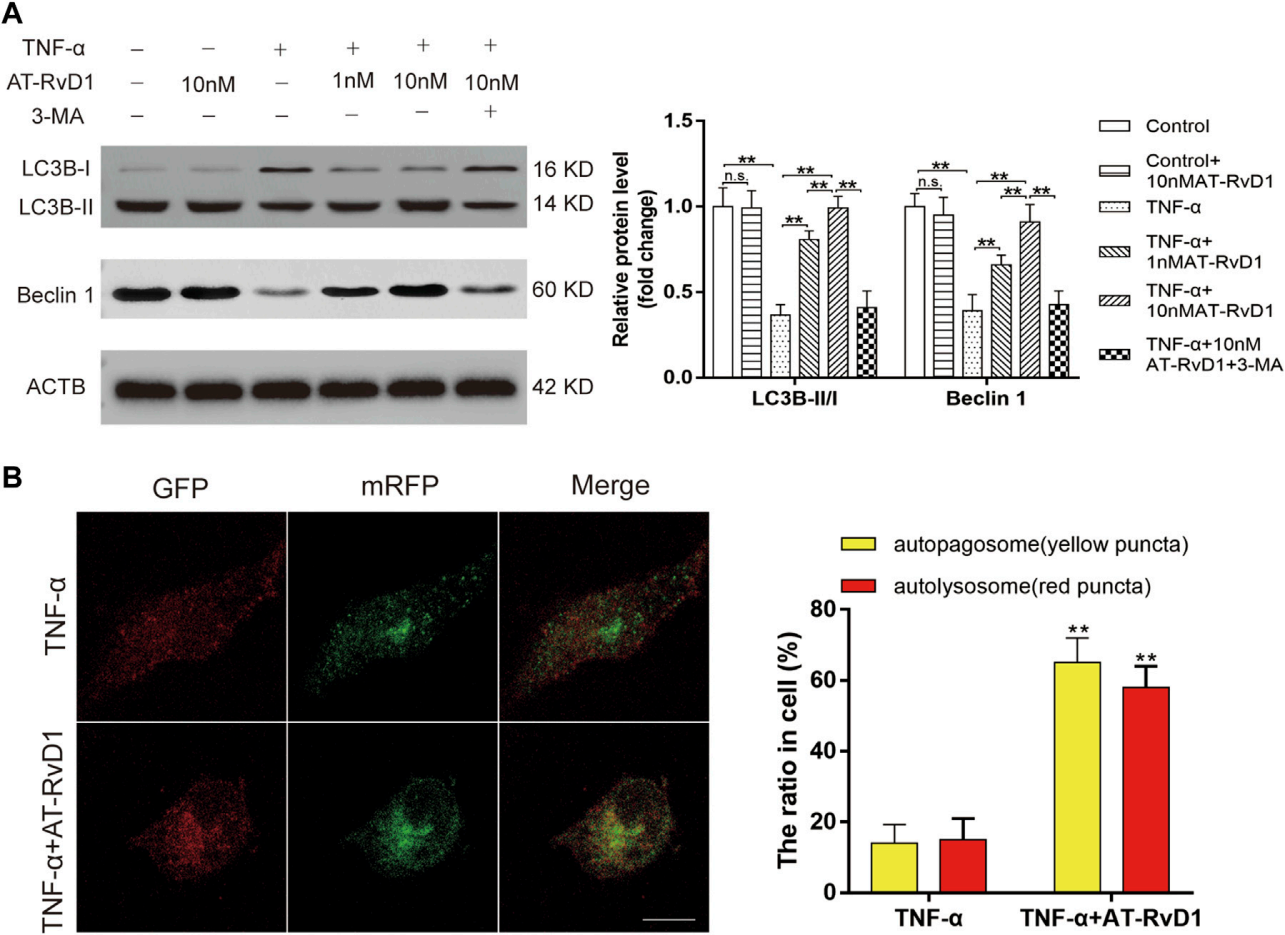
The effect of administration of AT-RvD1 on the activation of autophagy in TNF-α-stimulated microglia **(A)** Representative photomicrographs of western blot bands and densitometric analysis for the expression of Beclin1, LC3B-II, and LC3B-I proteins in the different groups (*n* = 5/group). **(B)** Photomicrographs illustrating immunofluorescence of microglia transfected with GFP-mRFP-LC3B adenovirus (*n* = 3/group). The GFP-mRFP-LC3B puncta were visualized under fluorescent confocal microscope used as an indicator for autophagosome-lysosome fusion. Red puncta indicate fused autophagosome with lysosome and the yellow puncta indicate unfused autophagosome (scar bars = 25 µm for figures). Data were represented as mean ± standard error of mean (SEM). ∗*p* < .05. ∗∗*p* < .01. n. s. means no significant difference between independent experiments.

The concurrent increase in the level of autophagy-related proteins Beclin1 and the LC3B-II/I ratio showed a blockage of autophagic flux in primary cultured microglia cells after treatment with TNF-ɑ (Figure 3A). AT-RvD1 dose-dependently led to accumulation of Beclin1 and downregulated the LC3B-II/I ratio compared to that with TNF-ɑ alone in primary microglia. Moreover, compared with TNF-ɑ stimulation, AT-RvD1 enhanced the red punctuation/aggregation after mRFP-GFP-LC3 lentivirus transfection (Figure 3B). Next, we utilized the autophagy blocker 3-MA to assess the autophagic changes in TNF-α-stimulated microglia. Additionally, the results of quantification showed that 3-MA reversed the marked increase in the expression of Beclin1 and the LC3B-II/I ratio induced by AT-RvD1 (Figure 3A). Thus, these results further strengthen our earlier observation that AT-RvD1 is likely to promote autophagosome maturation and autophage-related protein formation, and treatment with 3-MA can decrease the AT-RvD1-mediated conversion.

### AT-RvD1 exerted neuroprotective effects and suppressed neuroinflammation by modulating the NLRP3 signaling pathway

**FIGURE 4:**
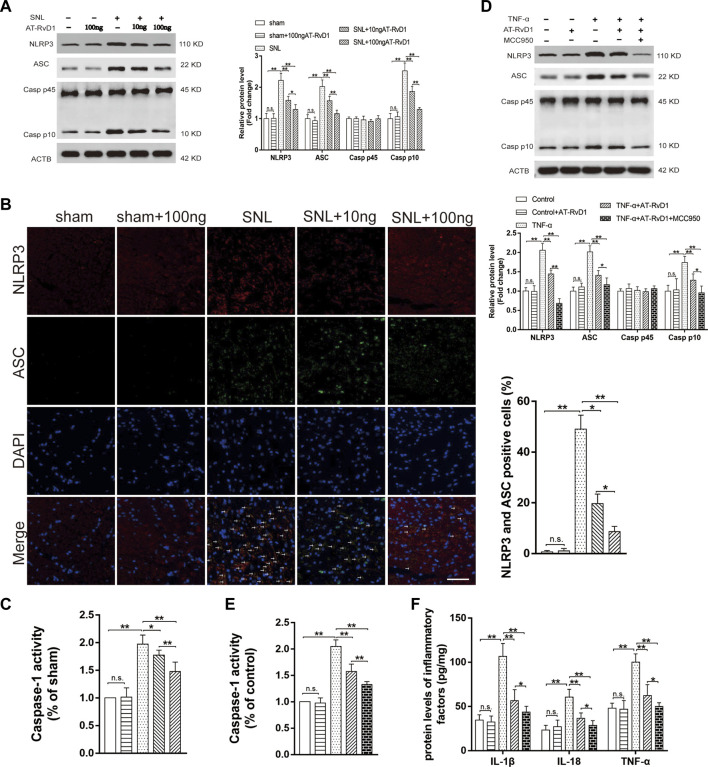
Effect of AT-RvD1 on NLRP3 inflammasome expression and inflammatory responses **(A)** Representative photomicrographs of western blot bands and densitometric analysis for the expression of NLRP3, ASC, Caspase-1 P10, and Caspase-1 P 45 proteins in the spinal cord dorsal horns of rats with spinal nerve injury (*n* = 5/group). **(B)** The activation of NLRP3 inflammasome was assessed by immunofluorescence, and the colocalization of NLRP3 and ASC subunits represented the NLRP3 inflammasome activation (*n* = 3/group) (scar bars = 60 µm for figures). **(C)** The comparisons of the Caspase-1 activity in different treated groups (*n* = 6/group). **(D)** Representative photomicrographs of western blot bands and densitometric analysis for the expression of NLRP3, ASC, Caspase-1 P10, and Caspase-1 P45 proteins in cultured microglia in different groups (*n* = 5/group). **(E)** The comparisons of the Caspase-1 activity in different treated groups (*n* = 6/group). **(F)** Enzyme-linked immunosorbent assay for the quantitative analysis of IL-18, IL-1β and TNF-α on inflammatory responses in the microglia (*n* = 6/group). Data were represented as mean ± standard error of mean (SEM). ∗*p* < .05. ∗∗*p* < .01. n. s. means no significant difference between independent experiments.

We investigated the anti-inflammatory role of AT-SPMs on the activation of NLRP3 inflammasome-associated proteins in the NP model. Western blotting images and results of quantification of the expression showed that NLRP3, ASC, and Caspase-1 P10 were activated in the spinal cord dorsal horns after SNL surgery (Figure 4A). Treatment with AT-RvD1 reversed the increase in the levels of NLRP3, ASC, and Caspase-1 P10 induced by SNL surgery (Figure 4A). To better demonstrate the effect of AT-RvD1 on activation of NLRP3 inflammasome, the colocalization of NLRP3 and ASC subunits was analyzed (Figure 4B). The data showed that the combination of NLRP3 and ASC was significantly enhanced in the SNL group, while reduced after AT-RvD1 treatment dose-dependently, manifesting that AT-RvD1 blocked NLRP3 inflammasome activation in SNL model rats. Then the Caspase-1 activity was evaluated in different groups (Figure 4C). It was significantly increased after SNL and reduced after AT-RvD1 dosing.

To further confirm the decreases in the NLRP3 inflammasome protein as a consequence of the effects of AT-RvD1, we used the selective inhibitor of NLRP3, MCC950, in the TNF-ɑ-stimulated primary microglia (Figure 4D–F). The expression of the NLRP3 inflammasome complexes, the activity of caspase1 and its master downstream regulators (IL-18, IL-1β, and TNF-ɑ) was lower in the TNF-α-stimulated microglia than in the supernatant without TNF-α stimulation. Additionally, compared with TNF-α group, the TNF-ɑ+AT-RvD1 group showed a decrease in the expression of NLRP3 inflammasome components, Caspase-1 activity and in the secretion of proinflammatory cytokines, respectively. When cells were pretreated with MCC950, most of the effect of TNF-α stimulation was drastically decreased in the primary microglia compared with that in the TNF-ɑ+AT-RvD1 group. Thus, administration of AT-RvD1 mediated the inflammatory response through inactivation of the NLRP3 inflammasome in the TNF-ɑ-stimulated microglia.

### AT-RvD1 alleviated NLRP3 inflammasome activation *via* upregulating autophagy in NP

**FIGURE 5:**
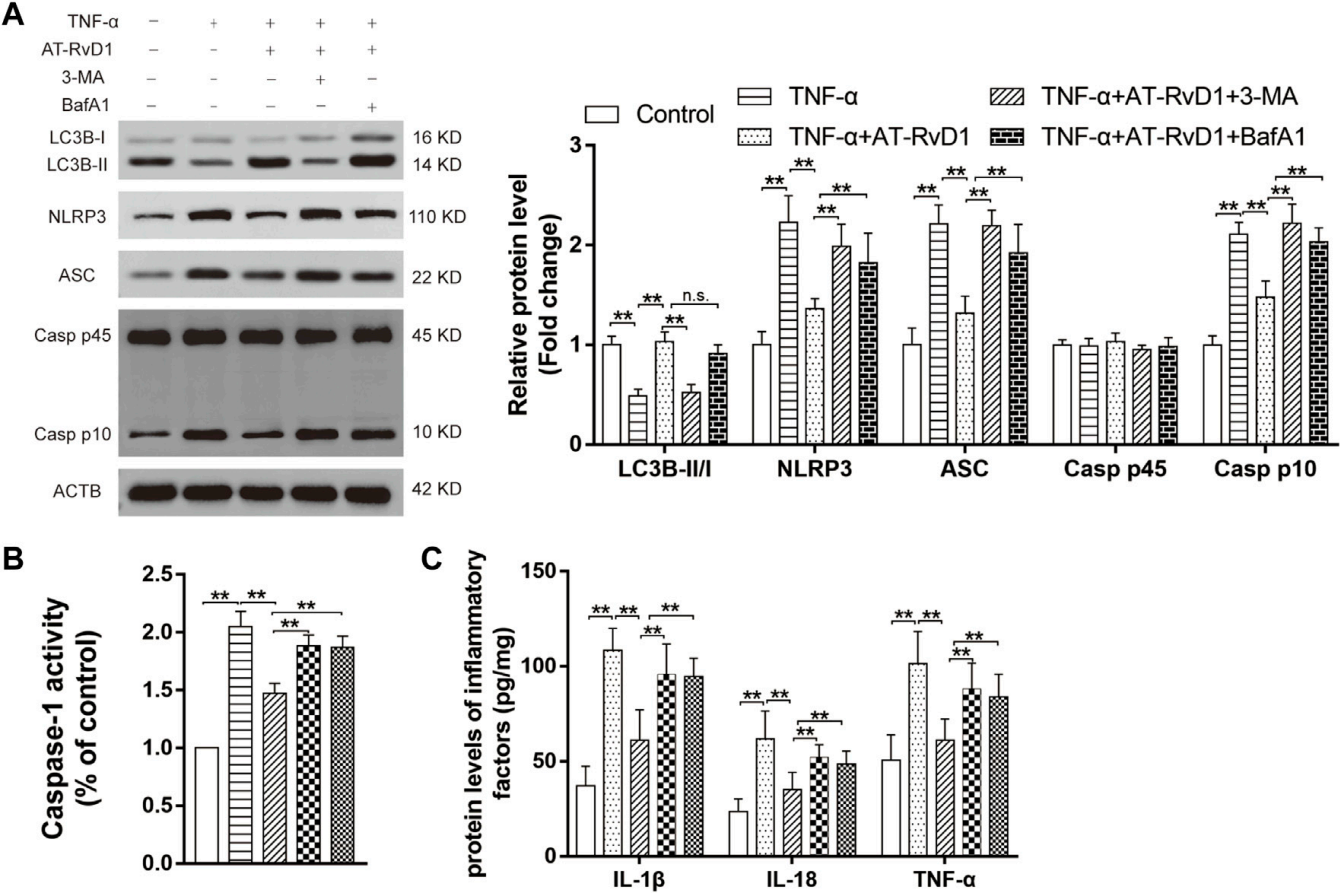
Correlation between autophagy and NLRP3 activation in a model of TNF-α-induced inflammation **(A)** Microglia stimulated with TNF-α were treated with AT-RvD1, 3-methyladenine (3-MA), and bafilomycin A1 (BafA1) in complete medium according to different groups (*n* = 5/group). The cell lysates were collected and subject to immunoblotting with specific antibodies against NLRP3, ASC, Caspase-1 P45, and Caspase-1 P10. **(B)** The comparisons of the Caspase-1 activity in different treated groups (*n* = 6/group). **(C)** Enzyme-linked immunosorbent assay for the quantitative analysis of inflammatory cytokines, including IL-18, IL-1β and TNF-α, in microglial inflammatory responses (*n* = 6/group). Data were represented as mean ± standard error of mean (SEM). ∗∗*p* < .01. n. s. means no significant difference between independent experiments.

Co-treatment with AT-RvD1 and 3-MA resulted in reduction in LC3BII/LCBI (Figure 5A), whereas concomitant administration of BafA1 and AT-RvD1 did not show a further increase in the LC3B-II/I ratio in the microglia denying the functional route of the lysosomal degradation of AT-RvD1. Moreover, compared with treatment with AT-RvD1, treatment with 3-MA or BafA1 both reversed this trend by inhibiting the formation of NLRP3, ASC, and Caspase-1 P10 in TNF-α-stimulated primary microglia. Similarly, compared with treatment with AT-RvD1, treatment with BafA1 or 3-MA enhanced the Caspase-1 activity and the production of IL-18, IL-1β, and TNF-α (Figures 5B, C) in TNF-α-stimulated microglia. Thus, these results support the notion that the process of autophagy is involved in activation of the NLRP3 inflammasome and in subsequent inflammatory responses.

### AT-RvD1 decreased hyperalgesia-related behavior and suppressed NLRP3 signaling pathways by modulating autophagy

**FIGURE 6:**
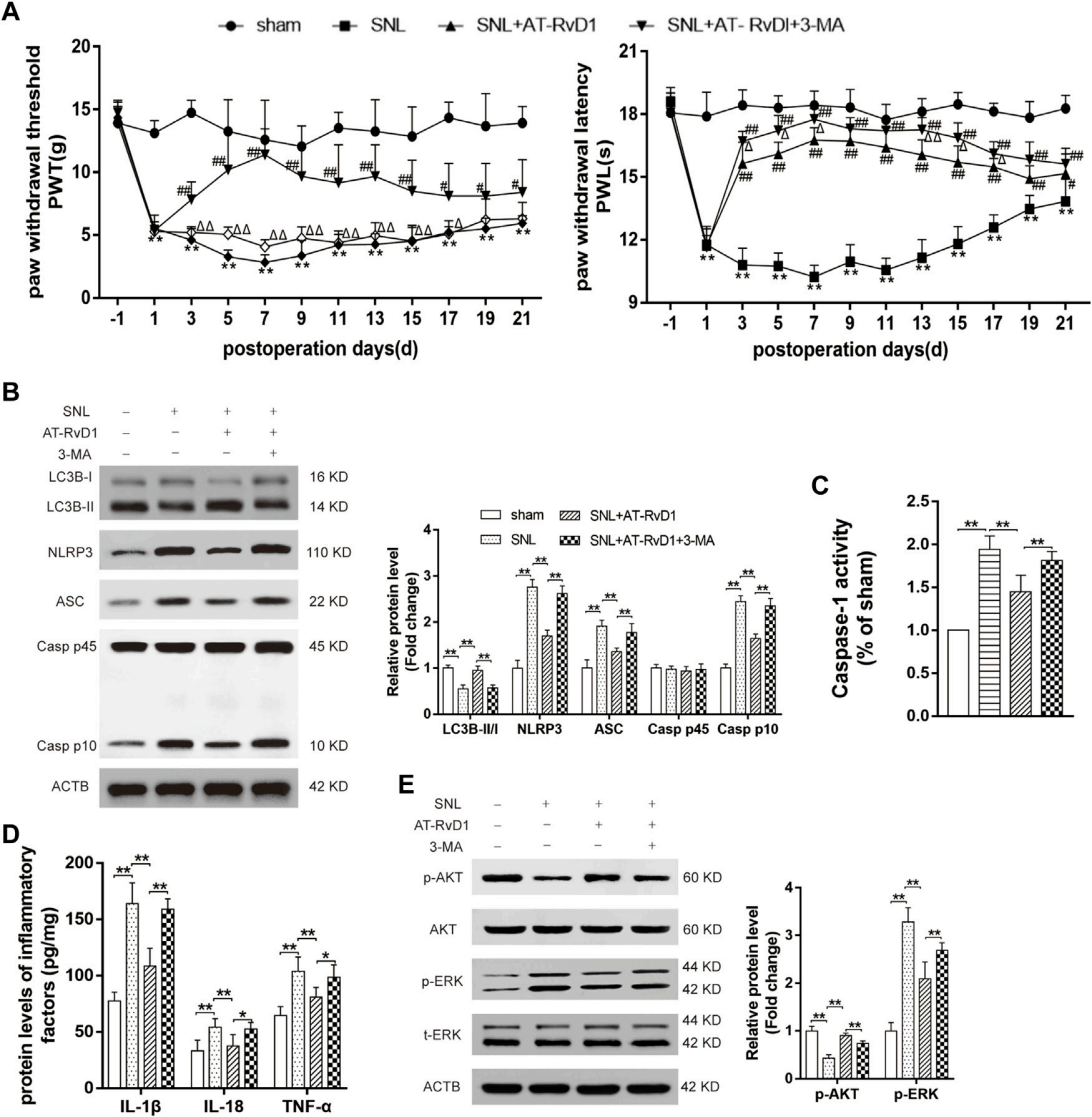
The function of autophagy on NLRP3 inflammasome pathway and hyperalgesia in a rat model of spinal nerve ligation-induced neuropathic pain **(A)** PWT and PWL were detected from preoperative day 1 to postoperative day 21 (*n* = 6/group). **(B)** Samples from the spinal cord dorsal horns of rats were collected to detect of the levels of LC3BI, LC3BII, NLRP3, ASC, Caspase-1 P10, and Caspase-1 P45 using western blot analysis (*n* = 5/group). **(C)** The comparisons of the Caspase-1 activity in different treated groups (*n* = 6/group). **(D)** Enzyme-linked immunosorbent assay for quantifying the expression of IL-18, IL-1β and TNF-α (*n* = 6/group). **(E)** Representative photomicrographs of western blot bands and densitometric analysis for the expression of p-AKT and p-ERK in the spinal cord dorsal horn of rats with spinal nerve injury (*n* = 5/group). Data were represented as mean ± standard error of mean (SEM). ∗*p* < .05 and ∗∗*p* < .01 compared with the sham group; ^#^ *p* < .05 and ^##^ *p* < .01 compared with the SNL group;^△^ *p* < .05 and ^△△^ *p* < .01 compared with the SNL + AT-RvD1 group.

To investigate the mechanism underlying the role of AT-RvD1 in regulating the autophagy process in the rat model of NP, we performed behavioral assessment after co-administration AT-RvD1 and 3-MA. Co-administration of AT-RvD1 and 3-MA reversed the trend of AT-RvD1 on the pain behavior after induction of nerve injury-induced NP (Figure 6A). Compared with the sham group, the AT-RvD1 group showed an increase in the LC3B-II/I ratio, which is a key indicator of the strength of autophagic activity. However, results of western blotting showed that cotreatment with the AT-RvD1 and 3-MA reduced the effect of AT-RvD1 on the LC3B-II/I ratio (Figure 6B). In addition, our results showed that treatment with AT-RvD1 suppressed the NLRP3 complexes proteins expressions, the Caspase-1 activity and decreased the expressions of proinflammatory cytokines (IL-18, IL-1β, and TNF-α) after nerve ligation, which were reversed by 3-MA to some extent (Figures 6B–D). As known, autophagy could be regulated by AKT and ERK signaling pathways (Rezq et al., 2021; Zhao et al., 2022). Therefore, we checked the changes of p-AKT and *p* -ERK protein expression (Figure 6E). The results showed that the protein expression of p-AKT was suppressed in SNL group, while AT-RvD1 treatment enhanced the expression, and reversed by the autophagy inhibitor. And the p-ERK protein expression gave the opposite trend. These data indicate that AT-RvD1 can decrease mechanical hyperalgesia-related behavior and improve the NLRP3 inflammatory response by mediating the autophagy process.

## Discussion

An imbalance of neuroinflammation in the nervous system is an important factor involved in NP, which may contribute to the development of persistent pain sensitization (Sommer et al., 2018). We recently validated that the activation of NLRP3 inflammasome and associated release of proinflammatory factors is linked to the prolongation of SNL-induced hyperalgesia. Here, we discussed whether the dysfunctional autophagic process could mediate the NLRP3 inflammasomes signaling pathway, which may be used as a potential therapeutic target for hyperalgesia. Moreover, these results showed a previously unknown mechanism underlying the unique activity of AT-SPMs and provided a novel therapeutic approach for NP.

Recent studies have shown that AT-SPMs, which exert a protective action in various neuroinflammatory diseases, can be used as novel therapeutic agents (Quiros and Nusrat, 2019; Inojosa et al., 2021). Our results are consistent with those reported previously (Wang et al., 2020; Wang et al., 2021) in that the administration of AT-RvD1 induces a progressive decrease in the PWT that persists for 3 weeks in a rat model of SNL-induced NP. Meanwhile, SNL-induced production of proinflammatory cytokines (IL-18, IL-1β, and TNF-ɑ) in the dorsal horn of the spinal cord was inhibited by AT-RvD1 in a dose-dependent manner. Recent studies indicate that activation of microglia release numerous chemical substances thereby leading to a neuroinflammatory response under NP conditions (Inoue and Tsuda, 2018). Therefore, we evaluated whether AT-RvD1 had beneficial effects in an *in vitro* inflammatory model established by culturing the primary microglia and stimulating them with TNF-ɑ to investigate neuroinflammation at the cellular biological level. Our results suggested that AT-RvD1 inhibited TNF-ɑ-stimulation induced accumulation of proinflammatory cytokines in primary cultured microglia. Overall, our data showed that SPMs exert a neuroprotective role *via* suppressing the accumulation of proinflammatory cytokines in an *in vitro* model of inflammation.

Previous studies indicate that autophagic process plays a crucial role in modulating microglia-mediated neuroinflammatory response (Plaza-Zabala et al., 2017; Choi et al., 2020). Recent studies indicate that dysfunction of the autophagy process plays an important role in the mechanisms underlying the biogenesis of neuralgia signal pathway (Chen et al., 2021). Additionally, our results showed that SNL decreased the levels of Beclin1 and ATG5 (the typical representative proteins of autophagic process) as well as the LC3B-II/I ratio (the *bona fide* autophagic marker), indicating that downregulation of autophagy activation was evident in NP. However, the administration of AT-RvD1 inhibited the SNL-induced decrease in the expression of autophagy-related proteins in a dose-dependent manner. Further, these results were supported by the results of our electron microscope observation that AT-RvD1 dose-dependently increased the staining of LC3B following SNL surgery. To further determine the cytological mechanisms involved in the neuroinflammatory process of NP, we explored the function of autophagy in an established *in vitro* model of inflammation, which is known to contribute to NP. Our results show that autophagic flux was impaired in TNF-ɑ-challenged microglia associated with a decrease in the of ratio of LC3B-II/I and the activation of the autophagy protein (Beclin1). Results of TEM and LC3B immunostaining showed a decrease in the number of autophagosomes in primary cultured microglia stimulated by TNF-ɑ. Our results showed that TNF-ɑ-stimulated microglia treated with AT-RvD1 showed an increase in the levels of ATG5 and Beclin1 as well as the upregulation of LC3B-II/I ratio. Addition of the autophagy blocker reversed the effect of AT-RVD1 on autophagic-related protein in TNF-ɑ-treated microglia. These results suggest that SPMs-mediated cytoprotection is dependent in part on a functional autophagy pathway. However, whether and how AT-RvD1 target microglia-mediated autophagy modulating the development of NP remains largely unknown thus far.

NLRP3 inflammasome is essential for the occurrence and development of diseases associated with a number of neurological conditions (Ising et al., 2019; Irrera et al., 2020; Zhou et al., 2021). The results of our study and previous studies (Wang et al., 2020; Wang et al., 2021) showed that intraperitoneal injection of AT-RvD1 dose-dependently downregulated the activation of the NLRP3 inflammasome complexes associated with improving the hyperalgesia behavior following the SNL surgery. Further, we extend the findings reported previously by comparing the outcome of the microglial inflammatory model, and our results showed that activation of NLRP3, ASC, and cleaved Caspase1 associated with an increase in the levels of proinflammatory factors were observed in cultured microglia after TNF-ɑ stimulation. Moreover, our results demonstrated that AT-RvD1 treatment weakened the induction of NLRP3 inflammasome accompanied by the secretion of IL-18, IL-1β, and TNF-ɑ *in vitro* after TNF-ɑ stimulation. In order to provided further evidence that this inflammasome is a major driver of neuroinflammation, we utilized the specific NLRP3 inhibitors (MCC950) in TNF-ɑ-stimulated microglia. Our results showed that the application of MCC950 increased the effect of AT-RvD1 on the downregulation of NLRP3 inflammasome complexes activation. On the basis of these data, we could identify the important role of the NLRP3 inflammasome complex as a key therapeutic biomarker of AT-SPMs in neuroinflammatory disease.

Recent studies have shown the protective role of autophagy in NLRP3 inflammasome activation and amelioration of intestinal inflammation in murine colitis models (Cosin-Roger et al., 2017b). Therefore, understanding the relationship between NLRP3 inflammasomes and autophagy processes is necessary to comprehend the mechanisms and designing possible treatments for neuroinflammatory diseases. Here, we further examined the effect of administration of the autophagy inhibitor, 3-MA, starting at 3 days after SNL on NLRP3 inflammasome complex of spinal cords and the mechanical allodynia behaviors. Blocking the autophagic activity using 3-MA reversed the AT-RvD1-mediated NLRP3 inflammasome inhibition and amelioration of algesia. Thus, we have reasons to speculate that AT-SPMs may exert a neuroprotective effect mediated by the autophagic process through negatively regulating the activation of the NLRP3 inflammasome complex. To support this hypothesis, we analyzed whether reduction in autophagy also increased the inflammatory reaction *in vitro* in presence of TNF-ɑ-stimulated primary cultured microglia. Inhibition of the formation of autophagosomes by 3-MA or BafA1 reversed the effect of treatment with AT-RvD1 by increasing the activation of the NLRP3 inflammasome complexes and the secretion of proinflammatory cytokines in TNF-ɑ-stimulated microglia. Similarly, as previous reported (Ba et al., 2019; Liu et al., 2021; Wang et al., 2022), in our study, AT-RvD1 meliorated activation of the NLRP3 inflammasome *via* induction of autophagy the regulated by AKT and ERK signaling pathways. And we will further explore the specific regulatory mechanism in the next experiments. Taken together, our data revealed that SPMs exert a neuroprotective effect in an autophagy-mediated NLRP3-dependent manner. However, based on the limited number of the experimental rats and molecular mechanism, the correction between autophagy and NLRP3 inflammasome in NP development was still further studied.

In summary, this present study highlighted the therapeutic function of SPMs-dependent neuroprotective effect on allodynia with an emphasis on the connection of novel regulation of cellular autophagic targets with and NLRP3 inflammasome pathways. The results of this study provide novel insight into the examination of neuroinflammation-related disorders with AT-SPMs as therapeutic interventions and create a basis for planned future efficacy trials.

## Data Availability

The raw data supporting the conclusion of this article will be made available by the authors, without undue reservation.
